# Myeloid-Derived Suppressor Cells in Lung Transplantation

**DOI:** 10.3389/fimmu.2019.00900

**Published:** 2019-04-26

**Authors:** Tobias Heigl, Anurag Singh, Berta Saez-Gimenez, Janne Kaes, Anke Van Herck, Annelore Sacreas, Hanne Beeckmans, Arno Vanstapel, Stijn E. Verleden, Dirk E. Van Raemdonck, Geert Verleden, Bart M. Vanaudenaerde, Dominik Hartl, Robin Vos

**Affiliations:** ^1^Lung Transplant Unit, Lab of Respiratory Diseases, Department of Chronic Diseases, Metabolism and Ageing (CHROMETA), KU Leuven, Leuven, Belgium; ^2^Universitätsklinik für Kinder-und Jugendmedizin, Tübingen, Germany

**Keywords:** myeloid-derived suppressor cells, blood, lung transplantation, allograft, chronic rejection, immunosuppression, infection, phenotypes

## Abstract

Myeloid-derived suppressor cells (MDSC) are a heterogeneous group of immune cells from the myeloid lineage. MDSCs expand in pathological situations, such as chronic infection, cancer, autoimmunity, and allograft rejection. As chronic lung allograft dysfunction (CLAD) limits long-term survival after lung transplantation (LTx), MDSCs may play a role in its pathophysiology. We assessed phenotype and frequency of MDSCs in peripheral blood from lung transplant recipients and its relationship to post-transplant complications and immunosuppression. Granulocytic (G)-MDSC were identified and quantified by flow cytometry of blood from 4 control subjects and 20 lung transplant patients (stable *n* = 6, infection *n* = 5; CLAD *n* = 9). G-MDSC functionality was assessed *in vitro* by their capability to block CD4 and CD8 T cell proliferation. More G-MDSC could be assessed using EDTA tubes compared to heparin tubes (*p* = 0.004). G-MDSC were increased in stable lung transplant recipients vs. non-transplant controls (52.1% vs. 9.4%; *p* = 0.0095). The infection or CLAD groups had lower G-MDSCs vs. stable recipients (28.2%*p* = 0.041 and 33.0%; *p* = 0.088, respectively), but were not different among CLAD phenotypes. G-MDSC tended to correlate with cyclosporine A and tacrolimus levels (*r*^2^ = 0.18; *r*^2^ = 0.17). CD4 and CD8 cells proliferation decreased by 50 and 80% if co-cultured with MDSCs (1:6 and 1:2 MDSC:T-cell ratio, respectively). In conclusion, circulating MDSCs are measurable, functional and have a G-MDSC phenotype in lung transplant patients. Their frequency is increased in stable patients, decreased during post-transplant complications, and related to level of immunosuppression. This study may pave the way for further investigations of MDSC in the context of lung transplantation.

## Introduction

From a transplant immunological point of view, graft acceptance is the fundamental element in allograft survival. Graft acceptance is realized by blocking the immune system with immunosuppression preventing host immune cells to recognized and attack the “non-self” donor (lung) tissue. Immune regulatory cells are thought to play a major role in the balance between graft acceptance and chronic rejection. Most attention has gone to natural and inducible FoxP3 positive regulatory T cells (Treg) (1). Immune regulation and graft acceptance, however, encompasses many more cells including regulatory B cells, regulatory dendritic cells and innate regulatory cells like the myeloid-derived suppressor cells (MDSCs), which were introduced 10 years ago by Gabrilovich et al., MDSCs were initially described as a heterogeneous group of immune cells from the myeloid lineage with a potent immune-regulatory activity (2). In the last few years, more insights into the nature and biological role of MDSCs have been reported and consequently MDSCs have emerged as a universal regulator of immune function in many pathologic conditions. MDSCs are known to expand in pathological situations such as chronic infection, cancer, transplant rejection and autoimmunity (3–5). Within the MDSC population, two main subgroups of cells were identified: granulocytic MDSCs (G-MDSCs) also nominated as polymorphomononuclear (PMN-MDSCs) and monocytic (M)-MDSCs. G-MDSCs are phenotypically and morphologically similar to neutrophils, whereas M-MDSCs resemble monocytes (6). Looking at the functionality of both M- and G-MDCS, the suppressive activity has been mainly attributed to arginine 1 (ARG1) and nitric oxide (NO) for M-MDSC and upregulation of reactive oxygen species (ROS) for G-MDSC (7, 8). Upregulation of ARG1, NO, and ROS are key mechanism to suppress T cell proliferation (9) and the production of IFNγ (10). Another hallmark is the upregulation of the transcription factor signal transducer and activator of transcription 3 (STAT3). STAT3, which functions as a signaling hub, integrating the different cues of the immunologic micro-environment (11, 12) regulates the expansion of MDSCs by stimulating myelopoiesis and inhibiting myeloid-cell differentiation. Further, it promotes MDSC survival by inducing the expression of cyclin D1, B-cell lymphoma XL (BCL-XL) and MYC (4). Within transplantation, MDSCs are involved in maintaining allogeneic acceptance in bone marrow, kidney and liver transplantation (13–16). Moreover, it has also been shown that commonly used immunosuppressive drugs can affect MDSC differentiation and functionality (17, 18). Our goal was to characterize phenotype (M-MDSC or G-MDSC) and frequency of MDSCs in lung transplant recipients. And consequently, to assess if MDSCs can serve as a potential new research target in the field of lung transplantation since chronic lung allograft dysfunction (CLAD), considered to be driven by an overactive T cell response, remains the most important factor limiting long-term survival after transplantation.

## Methods

### Patient Characteristics

This study included 20 lung transplant recipients and 4 healthy controls recruited at the University Hospitals Leuven (Belgium). All lung transplant recipients gave informed consent at time of listing for transplantation and routine blood sampling was approved by the University hospital (S51577). Relevant patient information retrieved from the clinical database included age, gender, type of transplantation, underlying disease, allograft ischemic time during transplantation, immunosuppressive dose, and trough levels, time post-transplant of blood sampling, time of death, infection information, and diagnostic criteria for CLAD and its phenotypes. Lung transplant recipients were selected according to their clinical status upon recruitment: 6 were considered stable, 5 recipients had an acute infection (2 CMV; 1 *Pseudomonas aeruginosa*; 1 Influenza + *E. coli*; 1 Influenza + *Aspergillus fumigatus*) and 9 were affected by different phenotypes of CLAD (5 BOS and 4 RAS cases). Blood of 15 individuals was used to compare Heparin vs. EDTA coated blood tubes (2 control, 3 Infection, 5 Stable, and 5 CLAD). The clinical status was assessed by an expert clinician (RV) according to current guidelines (19, 20).

### MDSC Characterization

**Figure 1:**
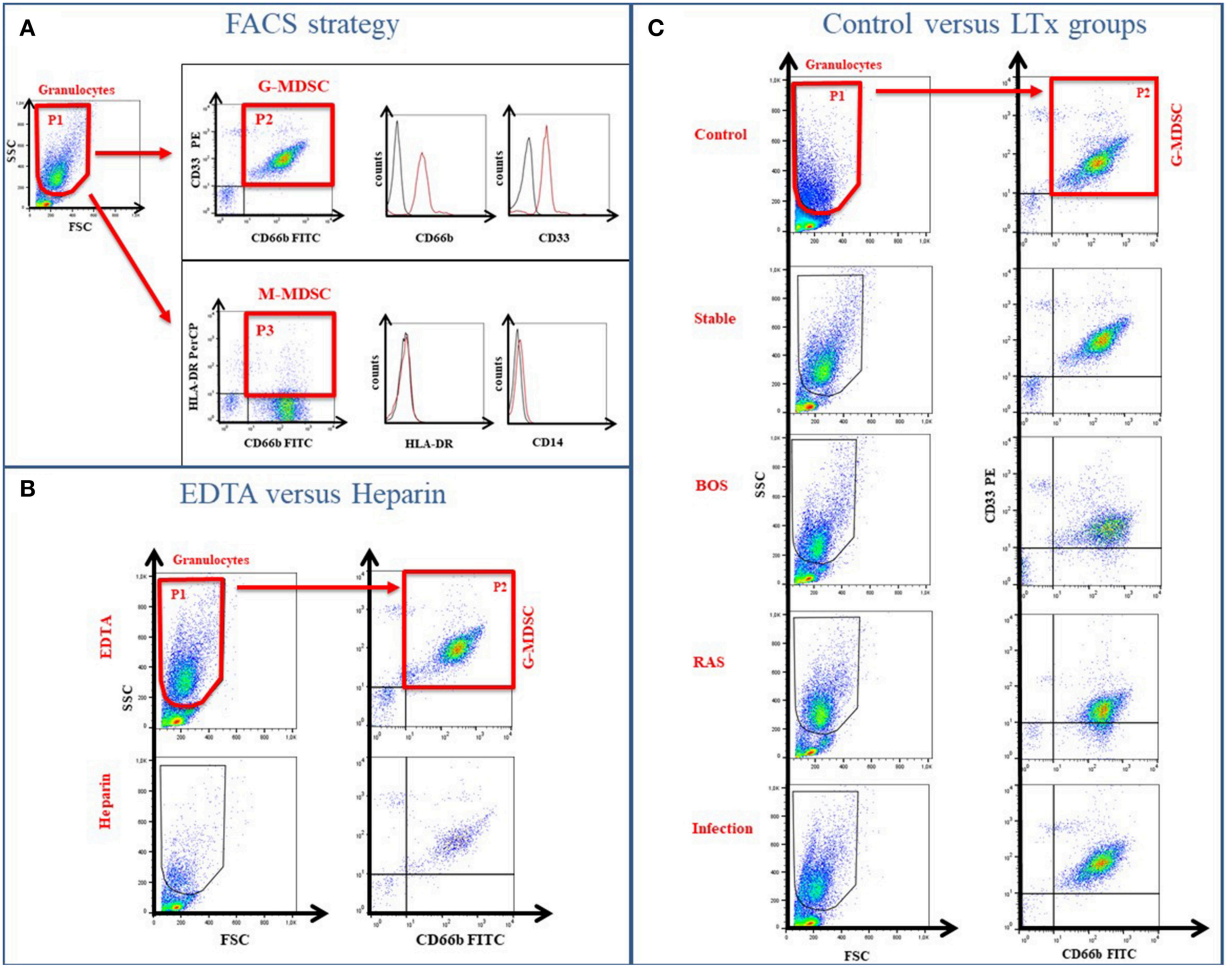
Gating Strategy to determine MDSC phenotype. **(A)** The low-density fraction of PBMC was stained with specific markers to differentiate between G-MDSCs (CD66b/CD33) and M-MDSCs (HLA-DR/CD14). **(B)** Different coatings of blood tubes (EDTA vs. Heparin) affect the MDSC cell numbers. **(C)** Exemplary FACS plots of the healthy controls and different LTx patient groups.

Peripheral blood was collected using EDTA and Heparin-coated tubes and samples were shipped to the Universitätsklinik für Kinder-und Jugendmedizin, Tübingen (Germany) at room temperature and analyzed within 24 h. MDSCs were characterized as previously described (21, 22). In brief, peripheral blood mononuclear cells (PBMCs) were isolated from whole blood by Ficoll density gradient centrifugation (Lymphocyte Separation Medium; Biochrom), washed with RPMI-1640 and cell viability was confirmed by trypan blue staining. The isolated PBMC, containing only low density granulocytes, were stained with specific antibodies for G-MDSC (CD66b-FITC, CD33-PE) and M-MDSC (CD14-FITC and HLADR-PerCP) (Miltenyi Biotec) and quantified by flow cytometry using a FACSCalibur (BD). G-MDSCs were phenotypically characterized as low-density fraction granulocytes CD33^+^CD66b^+^ cells (Figure 1). The percentage of G-MDSC was determined as ratio of CD33^+^CD66b^+^ cells (P2 in Figure 1) over total PBMCs containing the low density granulocyte fraction (P1 in Figure 1). Calculations were performed with BD CellQuest Pro analysis software and FlowJo V7.

### T-Cell Suppression Assays

**Figure 2:**
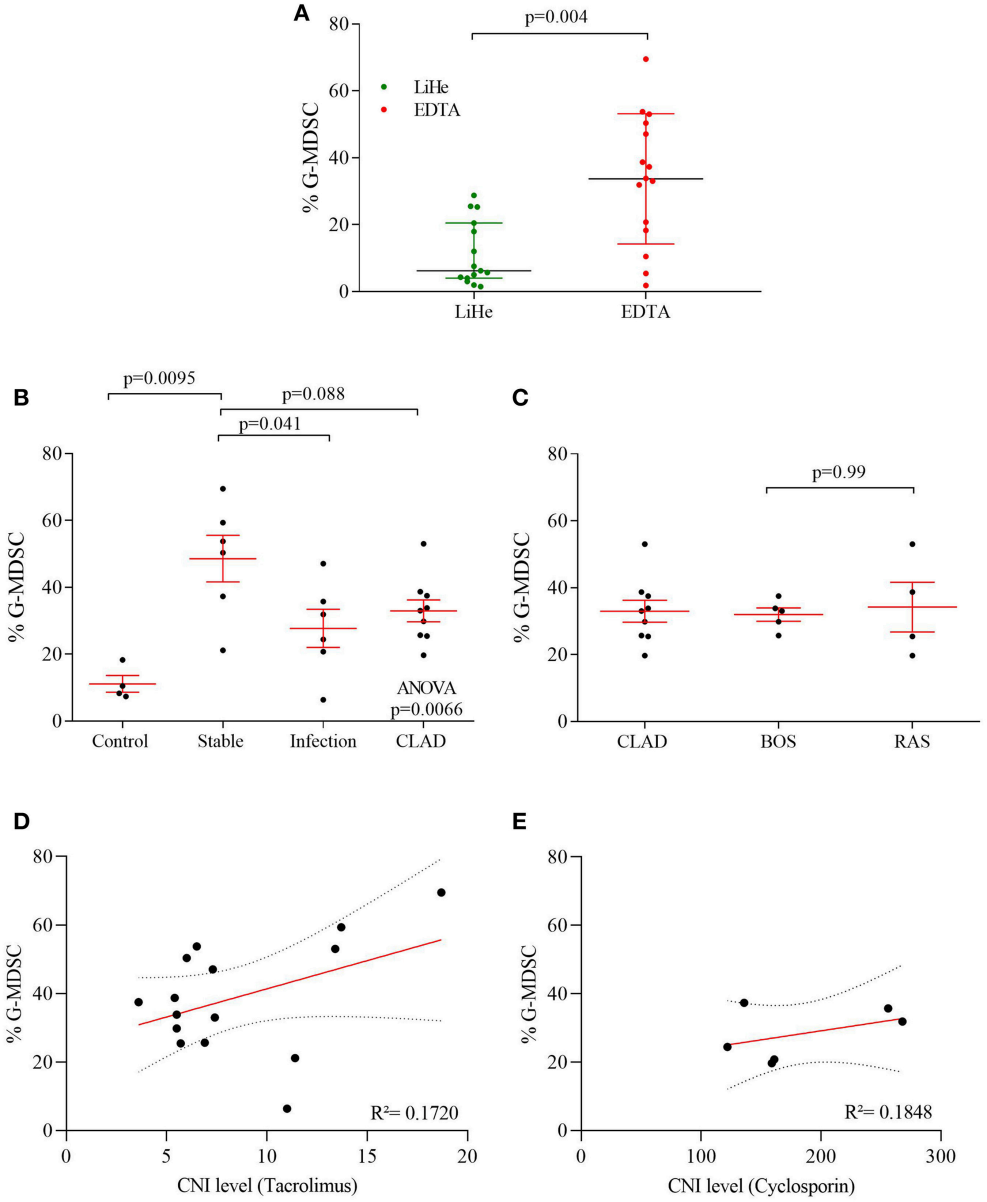
G-MDSC percentages measured in blood of lung transplant recipients and healthy controls. **(A)** the effect of LiHe vs. EDTA tubes on G-MDSC percentages; **(B)** G-MDSC in healthy controls and lung transplant recipients who were stable, had an infection or were diagnosed with CLAD; **(C)** CLAD sub-phenotypes in BOS and RAS. **(D,E)** G-MDSC correlated with CNI level of the patients.

The MDSC functional assay assessed T-cell suppression (both CD4 and CD8) by isolated MDSC (Figure 2) (23). MDSCs were isolated from blood of 2 lung transplant recipients, 1 stable and 1 with CLAD (BOS), using anti-CD66b and anti-FITC magnetic microbeads with the autoMACS®Pro Separator (Miltenyi Biotec) according to manufacturer's instructions. CD4^+^ and CD8^+^ T cells were isolated using CD4 and CD8 antibody (BD Pharmingen) combined with anti-FITC magnetic microbeads and autoMACS®Pro Separator (Miltenyi Biotec). Isolated CD4 or CD8 cells were labeled with CFSE dissolved in RPMI-1640, supplemented with 10% heat-inactivated human serum, 2 mM glutamine, 100 IU/ml penicillin, and 100 mg/ml streptomycin and 60,000 cells were plated per well in a 96-well microtiter plate. Cells were further stimulated with 100 U/ml IL-2 (R&D Systems) and 1 μg/ml OKT3 (Janssen Cilag). Different numbers of G-MDSCs were added to obtain an MDSC:T-cell ratio 1:6 and 1:2 and incubated for 3 days in a humidified chamber at 37°C and 5% CO_2_. After incubation, cells were harvested and CFSE-fluorescence intensity analyzed by flow cytometry to determine T-cell proliferation. Proliferation was calculated as the ratio of the divided cells (P1 to P5) over all cells (P0 to P5) with control T cells as reference value.

### Statistical Analysis

Qualitative variables are expressed as absolute numbers and percentages. Normally distributed quantitative variables are expressed as mean and standard deviation; non-normally distributed variables are expressed as median and interquartile range (25–75 percentile). Demographic and clinical variables of patients were compared using the chi-square test for qualitative variables or Fisher's exact test when one of the expected effects was <5. Normally distributed quantitative variables were compared using one-way ANOVA test; non-normally distributed quantitative variables were compared using the Kruskal-Wallis test. One-way ANOVA Test was used to compare MDSCs counts between groups. Linear regression was used for investigating the interaction of MDSC% and immunosuppressive trough levels. Data were analyzed using Graph Pad prism 7.0 software (San Diego, CA, USA).

## Results

Clinical characteristics of study participants are included in Table 1.

**Table 1:** Characteristics of lung transplant patients.

	**All**	**Stable**	**Infection**	**CLAD**	***p***
	***n* = 20**	***n* = 6**	***n* = 5**	***n* = 9**	
Age, median (IQR)	55 (32–60)	51.5 (20–60)	58 (36–62)	54 (39–57)	0.65
Gender: Male, *n* (%)	9 (42.9)	2 (33.3)	2 (40.0)	4 (44.4)	0.17
**Diagnosis**, ***n*** **(%)**					
COPD	10 (50.0)	3 (50.0)	3 (60.0)	4 (44.4)	0.86
ILD	2 (10.0)	0 (0.0)	0 (0.0)	2 (22.2)	0.47
CF	5 (25.0)	2 (33.3)	1 (20.0)	2 (22.2)	1.00
Other	3 (15.0)	1 (16.7)	1 (20.0)	1 (11.1)	1.00
**Immunosupressive treatment**, ***n*** **(%)**					
CsA+AZA+P	2 (10.0)	1 (16.7)	0 (0.0)	1 (11.1)	1.00
CsA+MMF+P	2 (10.0)	0 (0.0)	2 (40.0)	0(0.0)	0.05
CsA+P	1 (5.0)	0 (0.0)	1 (20.0)	0 (0.0)	0.25
FK+AZA+P	5 (25.0)	1 (16.7)	1 (20.0)	3 (33.3)	0.82
FK+MMF+P	5 (25.0)	3 (50.0)	1 (20.0)	1 (11.1)	0.35
FK+P	4 (20.0)	1 (16.7)	0 (0.0)	3 (33.3)	0.41
FK	1 (5.0)	0 (0.0)	0 (0.0)	1 (11.1)	1.00
**Type of LTx**					
SSLT	18 (90.0)	6 (100.0)	5 (100.0)	7 (77.8)	0.48
SLT	2 (10.0)	0 (0.0)	0 (0.0)	2 (22.2)	
Survival post LTx (years), median (IQR)	7.0 (4.1–9.7)	5.6 (3.8–8.7)	4.3 (3.0–7.4)	7.5 (5.9–11.9)	0.18
Sampling time post LTx (months), median (IQR)	3.9 (0.9–6.6)	1.9 (0.7–5.1)	0.9 (0.5–4.7)	6.6 (4.8–9.3)	0.02

*IQR, interquartile range; COPD, chronic obstructive pulmonary disease; ILD, interstitial lung disease; CF, cystic fibrosis; CsA, Cyclosporine; FK, Tacrolimus; P, Prednisolone; LTx, Lung Transplantation*.

G-MDSC were present in the low-density fraction of PBMCs, based on physical (FSC/SSC) and flow cytometric characteristics (CD33^+^CD66b^+^ cells) (Figure 1A). M-MDSC, on the other hand, were not observed in the low-density fraction of PBMCs, based on physical (FSC/SSC) and cell surface marker characteristics (CD14^−^HLA-DR^−^) (Figure 1A).

Percentages of G-MDSC were increased when using EDTA tubes compared to using LiHe tubes (mean: 33.38% [range: 18.32–50.36] vs. 6.24% [4.02–20.53], *p* = 0.004) (Figures 1B, 2A). EDTA and LiHe tubes were equally (statistically not significantly different) distributed across the control and patient groups. G-MDSC were increased in stable lung transplant recipients vs. healthy control subjects (52.1% [33.3–61.9] vs. 9.4% [7.6–16.4], *p* = 0.0095) (Figures 1C, 2B). Lung transplant recipients with an infection or CLAD tended to have lower percentage of G-MDSC compared to stable recipients (28.2% [17.2–36.6], *p* = 0.041 and 33.0% [25.6-38.1], *p* = 0.088, respectively) (Figure 2B). Within CLAD patients, the proportion of G-MDSC were comparable in BOS (5 cases) and RAS (4 cases) (*p* = 0.99) (Figure 2C). G-MDSC percentages seemed to increase with increasing blood levels of the calcineurin inhibitors (Tacrolimus *r*^2^ = 0.17, *p* = 0.12; Cyclosporine *r*^2^ = 0.18, *p* = 0.39) used as immunosuppressive therapy, which however was not significant most probably due to the small sample size (Figures 2D,E).

**Figure 3:**
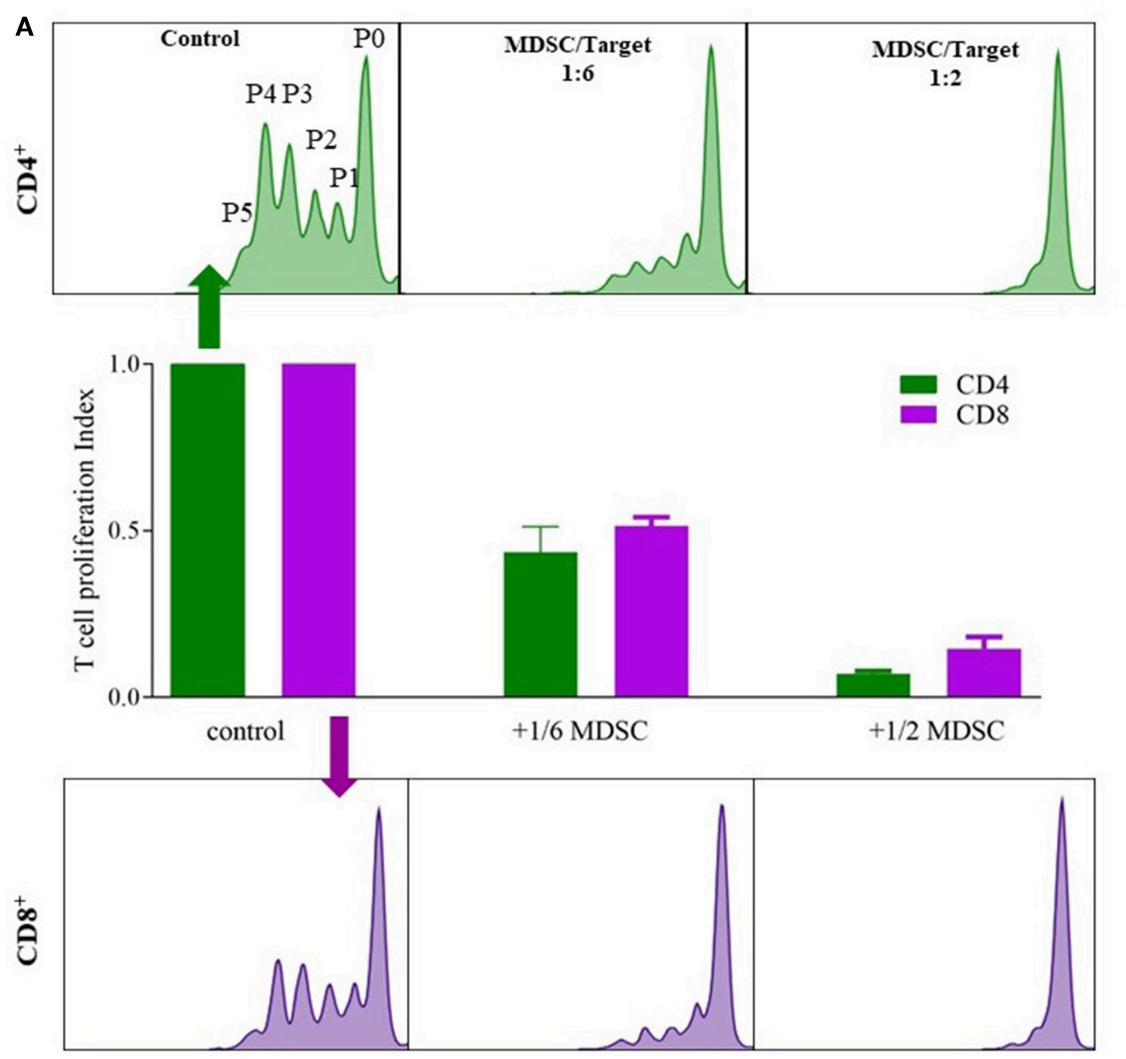
G-MDSCs isolated from lung transplant patients functionally supress T cell proliferation. The suppressive effect of CD66b^+^-MACS-isolated MDSCs (isolated from lung transplant recipients; 1 with CLAD and 1 stable) on CFSE labeled T cell CD4^+^ (green) and CD8^+^ (purple) proliferation. **(A)** Different ratios of MDSC vs. T cells (1:6 and 1:2) were assessed and compared with T cell proliferation without MDSCs. P0 represents undivided cells, P1 cells divided 1 time; P2 cells divided twice and so on. T cell proliferation ratio is portion of divided cells over all cells. The bar graphs represent the proliferation index compared to control conditions (*n* = 2).

G-MDSCs isolated from lung transplant patients effectively suppressed T-cell proliferation in a CFSE based polyclonal proliferation assay. The T-cell suppression assay was used as a proof-of-concept assay to demonstrate that G-MDSCs expanded in transplant recipient patients indeed represent a suppressive G-MDSC cell type and do not reflect myeloid cell populations with G-MDSC-like markers, but without T cell suppressive activities. Isolated patient G-MDSCs exhibited a strong suppressive function on T cell proliferation of about 50 and 80% with a 1:6 and 1:2 ratio of MDSC, vs. CD4^+^ or CD8^+^ T cells, respectively (Figure 3).

## Discussion

MDSCs were evaluated in lung transplant recipients and G-MDSC (CD33^+^/CD66b^+^) could be identified in the low-density fraction of PBMCs. G-MDSC (CD33^+^/CD66b^+^) cells also expressed CD11b, CXCR4 and HLA-DR^low^. The absence of CD14 expression confirmed their G-MDSC phenotype (Figure S1).

MDSCs are known for their role in immune regulation and allograft acceptance, and are involved in delayed graft rejection (17, 24, 25). Our data showed an expansion of G-MDSCs (not M-MDSCs) in stable lung transplant recipients and a decrease of G-MDSCs in patients with CLAD. Lung transplant recipients suffering from an infection also demonstrated a reduction in G-MDSCs, pointing to the fact that infection interferes with immune regulation and allograft acceptance. For example, it has been shown in mice that CMV infection impairs MDSC differentiation (26). CMV is a clinically relevant post-transplant pathogen, which is considered as a risk factor for later development of CLAD (27) Also in our study population, we found that recipients with diagnosed CMV within the infection group showed a lower G-MDSC percentage compared to the other patient groups (data not shown).

Furthermore, we evaluated the effect of immunosuppression on G-MDSCs: G-MDSCs showed a modest correlation with increasing CNI trough levels, a previously reported phenomenon (17, 28). Calcineurin inhibitors are indispensable in lung transplantation as efficient immunosuppressive drugs to block the immune response toward the allograft; hence, induction of MDSCs and their immunosuppressive function might be a part of their mechanism of action. It has been shown in a mouse skin transplant model that mechanistically, CsA treatment enhances the expression of indoleamine 2,3-dioxygenase (IDO) and thereby induces the suppressive activities of MDSCs in allograft recipients (29). Since the myeloid compartment consists of many different cell types with often overlapping phenotypic markers, we wanted to assess if the G-MDSCs, isolated from our lung transplant population, demonstrated suppressive effector properties. We confirmed that G-MDSCs did exert CD4^+^ and CD8^+^ T cell suppression in two independent patient samples. Due to the low number of replications, we can only speculate that in the setting of transplant immunology, G-MDSCs would act upstream of T cells to induce a cascade of peripheral tolerance toward the graft tissue. Challenging from a technical standpoint was the difference observed between the Lithium-Heparin and EDTA coated blood-drawing tubes used for PBMC isolations and the resulting differences in G-MDSC. At this point, we speculate that EDTA, as an iron chelator, inhibits cell degranulation, and may be the reason why more G-MDSC can be measured when using EDTA coating compared to Lithium-Heparin, at least in our experimental settings. However, it is important to mention that in a study by Pallet et al., the opposite effect, increased G-MDSC counts in Heparin vs. EDTA tubes, has been observed (30), which thus needs further investigation.

There are several limitations to our study. As a pilot study, the number of studied patients is limited. Furthermore, there are several confounding factors such as the heterogeneity of patient characteristics, differences in immunosuppressive therapy, use of azithromycin, different blood sampling tubes and different timings of sampling after lung transplantation.

However, our findings remain interesting, and may warrant more in-depth research on the role of G-MDSCs in lung transplantation. In our opinion, elucidating the functional hierarchy of immune regulatory cells in the context of transplant tolerance/rejection is of importance to understand graft acceptance. We believe that the up-stream suppressive activity of G-MDSC may be an intriguing starting point to dissect this highly complex interconnected immune regulatory system consisting of Treg, Bregs, Mregs, and other cell types.

## Ethics Statement

This study included 20 lung transplant recipients and 4 healthy controls recruited at the University Hospitals Leuven (Belgium). All lung transplant recipients gave informed consent at time of listing for transplantation and routine blood sampling was approved by the University hospital (S51577).

## Author Contributions

TH and ASi performed this study from sampling, analyzing, writing, and submitting the paper. ASi performed the MDSC FACS analysis, the *in vitro* MDSC proliferation test and the writing of the paper. JK, ASa, and SV helped in organizing the sampling and writing of the paper. DH helped in writing of the paper. BS-G, HB, AV, and AVH helped by searching for the clinical patient information and helped preparing the data. DV was the surgeon performing the lung translations and critical evaluated the manuscript. GV and RV were the clinicians performing the daily care taking of the patients and critical evaluated the manuscript. BV and RV designed and funded the study.

### Conflict of Interest Statement

The authors declare that the research was conducted in the absence of any commercial or financial relationships that could be construed as a potential conflict of interest.

## Supplementary Material

Figure S1 Representative dot plots and histograms of surface marker profiling of G-MDSCs isolated from lung transplant patients. MDSCs were analyzed in peripheral blood mononuclear cells (PBMCs) isolated from low density fraction of whole blood after Ficoll density centrifugation. Lung transplant MDSCs exhibited characteristic G-MDSC phenotype of CD33b^+^CD14^−^cells (**left** panel, also see Figure 1) and also expressed CXCR4 (**right** panel). Histograms show individual surface marker staining (red) in comparison to unstained control (black) for CXCR4.
