# Supplementary figures and images for: Myeloid-Derived Suppressor Cells in Lung Transplantation
Source: Front Immunol. 2019 Apr 26;10:900. doi: 10.3389/fimmu.2019.00900 (PMC6497753; doi:10.3389/fimmu.2019.00900)

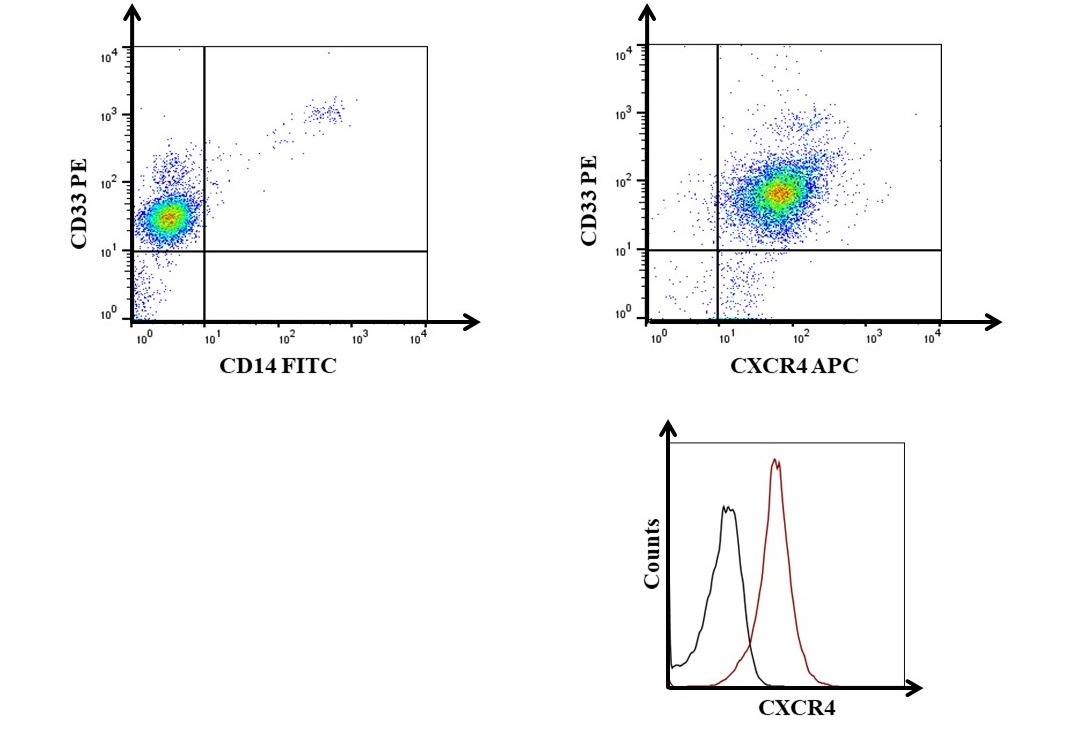

Supplement: Figure S1 — Representative dot plots and histograms of surface marker profiling of G-MDSCs isolated from lung transplant patients. MDSCs were analyzed in peripheral blood mononuclear cells (PBMCs) isolated from low density fraction of whole blood after Ficoll density centrifugation. Lung transplant MDSCs exhibited characteristic G-MDSC phenotype of CD33b+CD14−cells (left panel, also see Figure 1) and also expressed CXCR4 (right panel). Histograms show individual surface marker staining (red) in comparison to unstained control (black) for CXCR4. [file Image_1.JPEG]
